# Intensive smoking cessation treatment as an adjunct to concurrent psychotherapy: study protocol for a randomized controlled trial

**DOI:** 10.1186/s13063-025-08781-2

**Published:** 2025-03-03

**Authors:** Esra Teresa Sünkel, Alla Machulska, Marie Neubert, Tim Klucken

**Affiliations:** Department of Clinical Psychology and Psychotherapy, University of Siegen, Obergraben 23, Siegen, 57072 Germany

**Keywords:** Smoking cessation, Nicotine dependence, Cognitive behavioral therapy, Short-term treatment, Adjuvant treatment options, Substance use disorders

## Abstract

**Background:**

Tobacco use is globally recognized by the World Health Organization as the foremost risk factor for premature mortality. Individuals with mental disorders exhibit a notably heightened dependence on tobacco, approximately twice as high as that of the general population. The long-term effects of nicotine consumption include an exacerbation of depressive symptoms and a decline in mental health, which can be considered additional risk factors for the vulnerable population of smokers with preexisting mental disorders. Successful smoking cessation is associated with an increase in mental health, comparable or superior to that of pharmacological antidepressant treatments. However, smoking is frequently disregarded within the realm of psychotherapeutic care, unlike in the treatment of other substance use disorders. Smoking may hinder patients’ recovery and responsiveness to psychotherapy, potentially distorting improvements in symptom severity or negative affect. Integrating smoking cessation initiatives into standard psychotherapeutic interventions thus holds significant potential and may be considered essential for long-term mental health. The current study’s objective is to assess the potential of a guideline-based smoking cessation intervention within an outpatient psychotherapeutic setting concerning changes in smoking intensity, nicotine dependence, and mental health outcomes among patients with a mental disorder adjunct to concurrent psychotherapy. This trial aims to bridge the gap between the state of research in light of evidence of the positive effects of successful smoking cessation and the actual practical provision of care in Germany.

**Methods:**

The effects of an intensified smoking cessation intervention versus a treatment-as-usual (TAU) control intervention on smokers’ smoking intensity, nicotine dependence, and mental health symptoms related to a primary mental disorder will be examined in a single-center randomized controlled trial in an outpatient psychotherapeutic clinic using variance analysis methods. In this case, TAU is referred to as regular psychotherapy sessions without any additional smoking cessation treatment. The smoking intervention consists of a common guideline-based cognitive-behavioral program supplemented by a digital health application. Secondary outcomes include patients’ motivation to quit, self-efficacy, and attitudes toward online interventions. Potential moderators or mediators will be investigated in exploratory analyses.

**Discussion:**

This study aims to elucidate the potential benefits of integrating smoking cessation interventions into standard psychotherapeutic treatment, akin to approaches used for other substance use disorders. While existing research highlights the positive impact of smoking cessation on mental health, its practical implications within the field of psychotherapy remain unclear. To address this gap, the current study examines the effectiveness of an intensive smoking cessation program alongside ongoing psychotherapy, focusing on both smoking status and mental health outcomes. By doing so, we aim to provide practical insights for psychotherapeutic providers regarding the integration of smoking cessation into comprehensive mental health care.

**Trial registration:**

Prospectively registered on ISCRTN on 01.05.2024, reference number ISRCTN12859609.

## Administrative information

Note: The numbers in curly brackets in this protocol refer to SPIRIT checklist item numbers. The order of the items has been modified to group similar items (see http://www.equator-network.org/reporting-guidelines/spirit-2013-statement-defining-standard-protocol-items-for-clinical-trials/).

**Table:** 

Title {1}	Intensive smoking cessation treatment as an adjunct to concurrent psychotherapy: study protocol for a randomized controlled trial
Trial registration {2a and 2b}	Registered on ISCRTN, reference number ISRCTN12859609, submitted 12/02/2024, published on ISCRTN 01/05/2024
Protocol version {3}	Protocol version 2, 28.01.2025
Funding {4}	University of Siegen
Author details {5a}	Esra Teresa Sünkel, Dr. Marie Neubert, Dr. Alla Machulska, and Prof. Dr. Tim Klucken Department of Clinical Psychology and Psychotherapy, University of Siegen, Obergraben 23, 57072 Siegen, Germany
Name and contact information for the trial sponsor {5b}	Department of Clinical Psychology and Psychotherapy, University of Siegen, Obergraben 23, 57072 Siegen, Germany. Lab
Role of sponsor {5c}	The sponsor had no role in the study design and will have no role in data collection and analysis, decision to publish, or preparation of the manuscript. The sponsor does and will not have ultimate authority over any of these activities.

## Introduction

### Background and rationale {6a}

Tobacco consumption, identified by the World Health Organization (WHO) as the leading global risk factor for premature mortality, results in approximately six million premature deaths annually [21, 48, 56]. In Germany, tobacco is primarily consumed through cigarette smoking [30]. Individuals with mental disorders exhibit particularly pronounced tobacco dependence, which is approximately twice as high as that of the general population [5, 49]. Moreover, smokers with mental disorders tend to smoke more heavily [9] and use tobacco as a coping mechanism for negative affect rather than for pleasure [53]. Consequently, smokers with mental disorders face increased health risks and mortality rates, with up to a 25-year reduction in life expectancy compared to the general nonsmoking population [13, 32, 55].

Apart from physical health consequences, nicotine, which is the primary alkaloid in tobacco responsible for its addictive properties, has detrimental effects on mental health outcomes. Compared to nonsmoking individuals, regular nicotine consumption is associated with negative impacts on quality of life [45] or sleep quality [27], an increased risk for the development of posttraumatic stress disorders after exposure to traumatic events [34], and increased rates of depressive and anxiety symptoms [11, 24].

Various theories aim to explain the high prevalence of smoking among individuals with mental disorders, such as self-medication theories [14, 22, 35], neurobiological [19, 37] or bidirectional theories [12, 31, 39]. The latter focuses on the bidirectional link between smoking and mental health by describing the cyclic process of the initial short-term antidepressant effect of nicotine, which eventually leads to the exacerbation of depressive symptoms over the long term. For individuals with mental disorders, this means that smoking can exacerbate their symptoms over time, and conversely, aggravated mental health symptoms may contribute to continued smoking.

Because of this interplay, prolonged nicotine consumption can be considered a hindering factor for patients’ recovery and responsiveness to psychotherapy, possibly distorting potential improvements in symptom severity, including negative affect. The improbability of achieving successful therapy for a primary mental disorder without first attaining abstinence from a psychoactive substance has been acknowledged in all other substance use disorders. In cases of patients with other substance use disorders undergoing outpatient psychotherapy, the professional code of conduct for psychotherapists in Germany mandates prioritizing abstinence within the initial 10 sessions [51]. Given this precedent and considering the evident bidirectional link between smoking and mental health outcomes, the absence of a similar guideline for nicotine addiction is noteworthy. In addition, recent meta-analyses have indicated that successful tobacco cessation has long-term positive effects on mental health, as indicated by reduced symptoms of depression and anxiety, as well as overall increased quality of life [13, 52], and that these positive effects are comparable or even superior to those following pharmacological antidepressant treatment [52].

This preceding discussion implies a potential imperative for comprehensive treatment approaches that address both smoking cessation and mental health management simultaneously, indicating the importance of tailoring interventions to tackle smoking within the specific population of smokers with mental disorders. Furthermore, it underlines the importance of integrating tobacco cessation initiatives into established psychotherapeutic intervention methods, thereby ensuring sustained therapeutic success over an extended period of time. However, research indicates significant shortcomings in meeting this imperative. Studies reveal that guideline-conformive recommendations, such as actively addressing and intervening in smoking behavior, are scarcely implemented among healthcare providers, particularly in patients with primary mental disorders [5]. There are no specifically tailored interventions for smokers with a mental disorder as a primary diagnosis in Germany. Currently, the national treatment guidelines for tobacco-related disorders suggest offering this specific population the same smoking cessation aids as the general population [5], such as pharmacological treatment (for an overview see [26]), cognitive behavioral therapy (CBT)-based interventions (e.g., [36]), smartphone-based self-help programs (e.g., [44]) or multimethod programs (e.g., [23]), among others. There are clinical consensus narratives suggesting the superiority of more complex and intensive programs for smokers with comorbid mental disorders regarding treatment success and long-term abstinence [38]. However, this assertion is based on clinical presumptions and requires further empirical investigation.

For the field of psychotherapy, the gap in implementing guideline-conform recommendations may be attributed, in part, to a gap in systematically evaluated studies on smoking cessation adjunctive to concurrent psychotherapy. Bridging this gap is crucial for a comprehensive understanding of mental healthcare, while exploring the adjunctive integration of smoking cessation alongside concurrent psychotherapy has the potential to enhance the healthcare landscape by offering clearer insights into effective joint treatment methods.

The current study’s rationale is to offer a distinct perspective on smoking cessation among individuals who currently undergo psychotherapy by evaluating the effects of an adjunctive smoking cessation treatment within psychotherapeutic care. Based on the literature regarding the optimal treatments for substance use disorders other than nicotine addiction, the current study adopted an intensive smoking cessation intervention approach, which combined face-to-face smoking cessation sessions with licensed therapists with a digital health application for smoking cessation. The aim of this study is to examine the possible treatment effects of adjuvant smoking cessation treatment. Here, in addition to assessing a treatment effect on nicotine dependence and smoking intensity, our study also sought to monitor and analyze the effects of smoking cessation treatment on changes in mental health outcomes related to the primary diagnosis of a mental disorder. This is motivated by the well-documented positive effects of smoking cessation on mental health outcomes [13, 52]. In addition, we aim to monitor possible mediating factors, such as motivation to quit or willingness to change, and other possible mediating factors associated with mental health, such as self-efficacy, to control for interference and for potential secondary analyses. Consequently, by examining the treatment effects of adjunctive smoking treatment, our study endeavors to contribute a novel perspective to integrate smoking cessation interventions into standard psychotherapeutic care. Given that smoking cessation encompasses not only physical health but also mental well-being and the long-term effects of psychotherapy, we anticipate that our findings will foster a heightened awareness of the importance of addressing smoking cessation within the context of comprehensive mental health care.

### Objectives {7}

We expect a treatment effect on variables regarding smoking behavior as well as mental health outcomes, as evidenced by a significantly greater reduction in nicotine dependence severity, smoking intensity, quality of life, subjective psychological well-being, and perceived impairment of mental health symptoms and depressive symptoms in patients in the experimental condition than in patients in the control condition. These changes are expected to become apparent both in the short-term (i.e., post-treatment) as well as the long-term (i.e., 6 weeks and 6 months follow-up) when compared to the baseline measures.

### Trial design {8}

The present research project demonstrates a single-center randomized controlled trial examining the impact of an intensive smoking cessation treatment, in conjunction with psychotherapy, compared to a control group receiving TAU on nicotine dependence and mental health symptoms related to the primary mental disorder among smokers attending an outpatient psychotherapeutic clinic. All participants included in the study are receiving current psychotherapeutic treatment. Given the study’s objective to enhance care for patients lacking regular treatment access, we chose to include a treatment as usual (TAU) control group for comparison. Eligible participants will be randomly assigned to either the experimental group (diagnostic session + regular psychotherapy sessions + intensive smoking intervention (a six-session smoking cessation program and a self-guided digital health application)) or the TAU group (diagnostic session + regular psychotherapy sessions). Post assessments will take place directly after treatment, whereas follow-up assessments on outcome measures will occur 6 weeks (follow-up 1) and 6 months (follow-up 2) after study completion.

## Methods: participants, interventions and outcomes

### Study setting {9}

The study will take place at the University of Siegen’s outpatient psychotherapeutic clinic.

### Eligibility criteria {10}

Participants must be at least 18 years old and exhibit harmful use of tobacco (F17.1 according to the ICD-10; [40]) or tobacco dependence (F17.2 according to the ICD-10) while also fulfilling the criteria for a treatment-relevant primary mental disorder. Inclusion criteria also include a participant’s interest in changing smoking behavior. An exclusion criterion is acute suicidality. The smoking intervention will be performed by trained clinical psychologists, while all psychotherapy sessions will be performed by licensed psychological psychotherapists.

### Who will take informed consent? {26a}

The informed consent of study participants is ensured through a comprehensive briefing at the initiation of the study (initial session) by the psychotherapist. After receiving and reviewing the information, participants’ consent will be obtained.

### Additional consent provisions for collection and use of participant data and biological specimens {26b}

Not applicable.

## Interventions

### Explanation for the choice of comparators {6b}

By comparing the intensified smoking cessation program adjuvant to conventional psychotherapy to treatment as usual for psychotherapeutic patients, we can assess the effectiveness and possible superiority of our approach in terms of smoking cessation success and an improvement in mental health symptoms.

Secondly, selecting psychotherapeutic sessions as the comparator group allows for a pragmatic comparison, reflecting the real-world conditions under which individuals with primary mental disorders are typically supported. Comparing with this standard treatment contributes to ensuring the generalizability of the results to clinical practice applications.

Finally, this choice of comparators enables a comprehensive assessment of the benefits of the intensified smoking cessation program compared to standard psychotherapeutic care. This investigation aids in deriving evidence-based recommendations for the most effective and targeted intervention for smokers with primary mental disorders.

### Intervention description {11a}

The intervention in the experimental group consists of a manual-based six-step smoking cessation program. This treatment is adapted from a continuously examined and widely adopted tobacco cessation program in Germany, which is the smoking cessation program developed by the *Arbeitskreis Tabakentwöhnung* (Smoking Cessation Working Group) at the University Hospital for Psychiatry and Psychotherapy in Tübingen. This program, outlined in the manual by Batra and Buchkremer [3] and recently updated [4], is CBT-based and designed for both group and individual settings. In this study, the setting will be individual. Over the course of six sessions, participants will be guided in preparing for and executing their smoking cessation, developing strategies to maintain abstinence, and preventing relapses. The sessions can be clustered into three phases, as shown in Table 1.

**Table 1:** Smoking intervention phases

Phase 1	Session 1	Abstinence preparation phase: focuses on motivating individuals and understanding their smoking habits
	Session 2	
Phase 2	Session 3	Cessation phase: provides specific strategies for quitting smoking and managing cravings
	Session 4	
Phase 3	Session 5	Stabilization phase: emphasizes building alternative behaviors, coping with potential relapse triggers, promoting overall health, and managing slip-ups and relapses
	Session 6	

Additionally, personalized recommendations will be provided that are tailored to each individual’s smoking behavior. A precise description of the individual sessions can be found in the manual provided by Batra and Buchkremer [4]. In the present research project, the initial program will be intensified by increasing the frequency of the sessions and by adding a self-help smoking cessation smartphone application to the program to address potential unique challenges within the vulnerable group of smokers with mental disorders. While the original program by Batra and Buchkremer [4] was designed with weekly contacts, we plan to intensify the program by providing two sessions instead of one session per week. The increase in session frequency will result in a 3-week intervention duration. As mentioned above, we additionally provide the participating patient with access to the digital health application *NichtraucherHelden®* (Sanero Medical GmbH, Stuttgart, Germany), which is a CBT-based self-help smartphone application that includes eight modules of psychoeducation, tasks, and relapse prevention. A nationwide, multicentric, parallel, randomized controlled trial recently provided further evidence for the feasibility and efficacy of this guideline-based smoking cessation app, showing doubled abstinence rates on a 7-day point prevalence in the intervention condition compared to the control condition [44].

### Criteria for discontinuing or modifying allocated interventions {11b}

The assigned intervention can be terminated upon the patient’s request, and participants may withdraw from the study for any reason. Researchers retain the authority to discontinue a patient’s participation in the study if their mental health condition worsens, such as through experiencing a psychotic episode, exhibiting an acute increase in suicide risk, or an indication for hospitalization.

### Strategies to improve adherence to interventions {11c}

Protocol adherence monitored by the authors of the study, who ensure that the manual-based intervention is conducted as intended and that all the data were collected as described.

### Relevant concomitant care permitted or prohibited during the trial {11d}

There is no specific concomitant care administered nor prohibited during the trial. All eligible participants are in weekly contact with psychological psychotherapists during the intervention period.

### Provisions for post-trial care {30}

There is no anticipated harm or compensation for trial participation.

### Outcomes {12}

Primary outcomes include severity of nicotine dependence, smoking status, and mental health symptoms. Nicotine dependence and smoking status are measured at baseline (diagnostic session; t_0_), post (t_1_; t_0_ + 3 weeks), at follow-up assessment 1 (t_2_; t_1_ + 6 weeks), and at follow-up 2 (t_3_ + 6 months). Changes in nicotine dependency and smoking habits over time are operationalized as follows:

- Mean score changes in the “Fagerström Test for Nicotine Dependence” [17],German version: [8]
- Mean score changes in the “Alcohol, Smoking, and Substance Involvement Screening Test” (ASSIST) subscale for tobacco [47, 54]
- Mean score changes in the measured carbon monoxide (CO) level in exhaled breath using the Smokerlyzer®
- Mean score changes in self-administered urine tests measuring cotinine content with a dichotomous outcome (positive/negative; cutoff 200 ng/ml; for an overview, see [7])

Changes in mental health symptoms related to mental disorders are measured by self-report questionnaires assessed at baseline (t_0_), post (t_1_; t_0_ + 3 weeks), follow-up assessment 1 (t_2_; t_1_ + 6 weeks), and follow-up assessment 2 (t_3_ + 6 months). The used questionnaires target general psychological well-being and perceived quality of life, symptoms associated with mental disorders, and depressive symptoms.

Specifically, changes in mental health status are operationalized as follows:

- Mean score changes in the “Beck Depression Inventory-Revised” ([BDI-II]; [6])
- Mean score changes in the “Brief Symptom Check List” ([BSCL]; [20])
- Mean score changes in the German version of the “WHO Quality of Life-BREF” ([WHOQOL-BREF]; [1])
- Mean score changes in the German version of the “WHO-5 Well-being Index” ([WHO-5]; [43] )

Secondary and exploratory analyses will investigate the impact of the treatment on other clinically relevant factors, alongside examining potential moderators or mediators, such as self-efficacy. For these analyses, we assess secondary outcomes that are measured at baseline (t_0_), post (t_1_; t_0_ + 3 weeks), follow-up assessment 1 (t_2_; t_1_ + 6 weeks), and follow-up 2 (t_3_ + 6 months). In addition to self-efficacy, secondary outcomes include motivation to quit and motivation to change as well as attitudes toward digital health applications. Specifically, the measures include:

- Changes in both general and smoking-related self-efficacy are operationalized as follows:
  1. Mean score changes in the German version “Generalized Self-Efficacy Scale” ([GSE]; [29])
  2. Mean score changes in the German version “Self-Efficacy Scale for Smoking” ([SESS]; [28])
- Changes in patients’ motivation are operationalized as follows:
  1. Mean score changes in the “Motivation to Stop Scale” ([MTSS]; [41])
  2. Mean score changes in the German version of the “University of Rhode Island Change Assessment Scale” ([FEVER]; [25])
  3. Mean score changes in the “Scale for Stages of Change” ([SOC]; [16])
- Attitudes toward digital health applications are measured using mean scores of the questionnaire “Attitudes toward Psychological Online Interventions–the APOI” ([APOI]; [46])

### Participant timeline {13}

Participation in the study for interested patients will be arranged by the treating psychotherapist and the study team. Interested participants will then participate in a telephone interview to determine eligibility and fulfillment of the inclusion criteria (*-t*_*1*_). Following telephone screening for a duration of 10–30 min, patients will be invited to the Psychotherapeutic Outpatient Center of the University of Siegen for the first smoking cessation session, during which informed consent will be obtained, and psychological symptoms will be assessed within the course of a diagnostic session (baseline, *t*_*0*_). After the diagnostic session, during which all baseline measures are assessed, the patients will be randomly assigned to either the experimental group or the active control group. The experimental group will receive a 6-step smoking cessation program and additional access to a smartphone-based self-help application. The duration of the intervention will be 3 weeks. Afterwards, patients will be invited to the clinic again to assess all measures of interest (*t*_*1*_). Six weeks (*t*_*2*_) and 6 months (*t*_*3*_) after completion, patients will be invited once again for follow-up assessments. The participants’ timelines and data assessment points are shown in Table 2. During the duration of the study period, patients in both the experimental group and the control group will continue the ongoing psychotherapy sessions as usual. The intervention is adjunctive to ongoing psychotherapy, and the timeline of the current study has no influence on the course of psychotherapy.

**Table 2:**
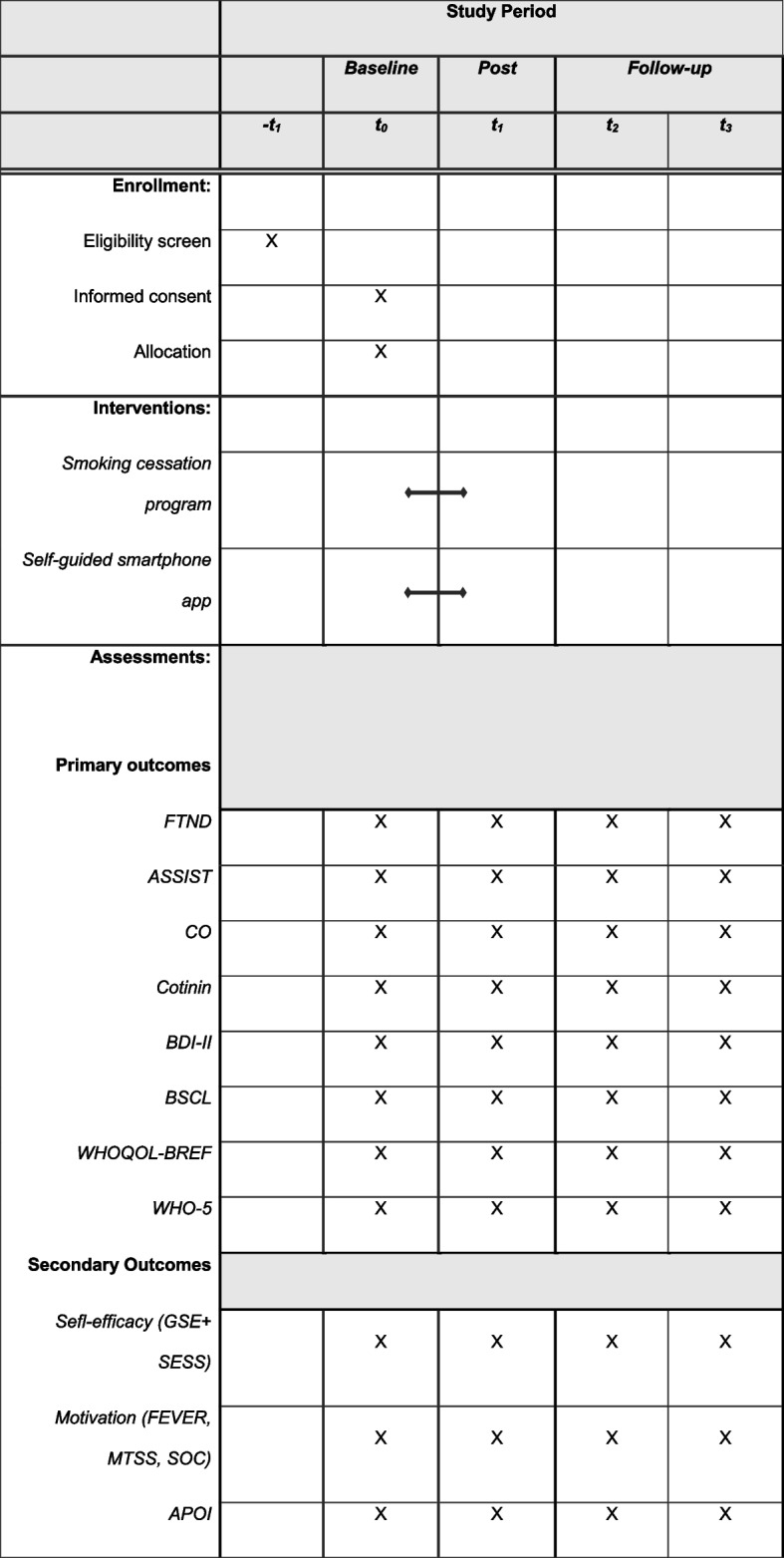
Participant timeline

### Sample size {14}

To estimate the required sample size, an a priori power analysis was conducted using G*Power 3.1.9.7 [18]. The primary hypothesis will be tested using a 2 × 4 mixed-design repeated-measures ANOVA with a power of 1 − *β* = 0.80 and a significance level of *α* = 0.05. This requires a sample size of approximately 64 participants to detect a moderate effect.

### Recruitment {15}

Participants will be recruited among patients identified as smokers in the outpatient psychotherapeutic clinic at the University of Siegen. If the inclusion rate does not meet the requirements, additional advertisements to enhance participation in this project will be installed by the research team. If necessary, the planned duration of the enrollment period of 1 year will be extended. Recruitment has started on May 1, 2024.

## Assignment of interventions: allocation

### Sequence generation {16a}

Participants will undergo random allocation to either the experimental or control group at a 1:1 ratio. Given the study design, participant or therapist blinding postallocation is not feasible.

### Concealment mechanism {16b}

No concealment mechanism, the groups are randomly allocated.

### Implementation {16c}

A research assistant or psychotherapist will communicate the allocation to patients and introduce them to either the intervention or the control group.

## Assignment of interventions: blinding

### Who will be blinded {17a}

The study follows an open-label design with partial blinding. Psychotherapists proposing study participation, along with research assistants handling participant communication, are blinded to group allocation. Data management tasks, such as data maintenance and transfer, are also performed in a blinded manner.

However, due to the nature of the intervention, neither the trained psychologists delivering the smoking cessation treatment nor the participants are blinded. Outcome assessment relies on participant-completed standardized questionnaires. Data analysis remains partially unblinded due to the necessity of including an allocation item for evaluating intervention outcomes.

### Procedure for unblinding if needed {17b}

Only the principal investigator and the trained psychologists delivering the smoking cessation have access to group allocation. The design is open label with only partial blinding of research assistants and psychotherapists, so no formal unblinding procedures are anticipated; however, any potential unblinding would be fully documented.

## Data collection and management

### Plans for assessment and collection of outcomes {18a}

In this study, training of assessors was facilitated by the research team to ensure standardized administration of the study instruments. The study instruments encompass a comprehensive array of assessments. The used questionnaires are all self-report instruments, and participants will be invited to complete the questionnaires digitally, meaning that all values will be transferred immediately to our database to promote data quality. The quality of the assessment will be ensured by having all participants complete the questionnaires independently during the diagnostic sessions in a quiet room without any distractions. All of the study’s instruments were chosen based on reliability and validity.

#### Primary outcome self-report questionnaires

The FTND [17], a ten-item scale with dichotomic items, is considered highly reliable and valid [42], (*rtt* = 0.88, *α* = 0.61). The scores can range from 0 to 10, while scores from 0 to 2 indicate no/very weak dependence, scores from 3 to 7 indicate moderate dependence, and scores of 8–10 indicate very high dependence.

The BDI-II is a self-report questionnaire assessing the severity of depressive symptoms across 21 symptom areas that has good internal consistency, *α* = 0.90–0.93, and its reliability can be considered good (*rtt* = 0.78; [6]). The total score can range from 0 to 63; scores below 9 indicate no evidence of depression, 9–13 suggest minimal depression, 14–19 indicate mild depression, 20 or above suggest moderate depression, and scores of 29 or above suggest severe depression.

The BSCL assesses an individual’s perceived impairment due to 53 physical and psychological symptoms, consists of nine subscales, and has been evaluated in numerous studies. The authors report good internal consistencies ranging from 0.71 to 0.85 and test-retest reliabilities ranging from 0.68 to 0.91, depending on the subscale [20].

The short version of the WHO Quality of Life Questionnaire (WHOQOLBREF) assesses the dimensions of physical well-being, psychological well-being, social relationships, and environment and is considered to have “good to excellent” psychometric properties [50], with internal consistencies ranging from 0.66 to 0.84 [57].

The WHO-5 is a psychometric tool used to assess an individual’s overall well-being and mental health and consists of five 6-step items. The German version has good internal consistency, *α* = 0.92, and very good reliability, *rtt* = 0.87 [10].

#### Primary outcome instruments targeting biomarkers

Carbon monoxide is assessed using a piCO™ Smokerlyzer® (Bedfont Scientific Ltd.). CO levels are objective markers of heavy smoking, and a value of 7.5 ppm in exhaled CO can be considered an appropriate cutoff [2]. Cotinine is assessed using nal von minden gmbH single rapid urine tests with a cutoff of 200 ng/ml, which is a commonly used cutoff for detecting nicotine exposure for up to 5 days [33].

#### Secondary outcome self-report questionnaires

The GSE measures generalized self-efficacy and has good reliability (*α* = 0.71–0.89; [29]). The unidimensional SESS measures smoking-related self-efficacy and consists of 9 items, demonstrating an internal consistency of *α* = 0.95 and test–retest reliability of *r* = 0.85 [28]. The FEVER measures stages of change and has good internal consistency, with Cronbach’s alpha ranging from 0.76 to 0.86 depending on the subscale [25]. For the SOC, a scale also measuring stages of change that varies from 0 (precontemplation) to 4 (maintenance), studies have reported a test–retest product-moment correlation of *r* = 0.78 [16].

The MTSS measures the motivation to quit smoking, and area under the receiver operating characteristics (ROC_AUC_) curves of the MTSS show good external validity (*ROC*_*AUC*_ = 0.64) [41].

The authors of the APOI report good internal consistency for depressive patients (Cronbach’s *α* = 0.77). In addition, convergent validity was tested by analyzing correlations of the APOI with an established instrument and was proven to be adequate, *r* = 0.74 [46].

### Plans to promote participant retention and complete follow-up {18b}

Participants will be informed about the study’s objective and the importance of completing the treatment and the follow-up evaluation, emphasizing the potential personal benefits of the treatment. Patients will receive a telephone call prior to each follow-up assessment to remind them of the date and encourage adherence to the program.

### Data management {19}

Data entry will be performed under the supervision of the research staff. The data will be electronically stored on a secure research server provided by the University of Siegen.

### Confidentiality {27}

All the data will be collected and stored in a pseudonymized manner (i.e., with a subject code) on a secure, access-restricted research server provided by the University of Siegen. Only anonymized data will be published. Subsequent linking of individuals to their data can only be performed with a datasheet, which will be securely stored and destroyed after the study is completed (but no later than 10 years).

### Plans for collection, laboratory evaluation, and storage of biological specimens for genetic or molecular analysis in this trial/future use {33}

Not applicable. Cotinine urine tests are evaluated immediately after the urine sample is provided and then disposed of using self-diagnostic test strips.

## Statistical methods

### Statistical methods for primary and secondary outcomes {20a}

Treatment effects on the primary outcomes regarding nicotine dependence and smoking intensity will be tested by computing mixed 2 (condition) × 4 (time) analysis of variance (ANOVA) for repeated measurements for each applied outcome variable. In the case of potential violations of the sphericity assumption, the Greenhouse–Geisser method will be applied.

It will be examined whether the experimental condition leads to higher abstinence rates at post- and follow-up assessments using chi-squared tests.

Treatment effects of the primary outcomes regarding mental health will be tested by computing mixed 2 (condition) × 4 (time) analysis of variance (ANOVA) for repeated measurements for each applied outcome variable, respectively. In the case of potential violations of the sphericity assumption, the Greenhouse–Geisser method will be applied.

Secondary and exploratory analyses testing for potential mediating variables will be conducted using regression analyses or structural equation modeling.

### Interim analyses {21b}

No interim analyses or formal stopping rules are planned for this study. The decision is based on the low-risk nature of the intervention, which involves behavioral support without invasive procedures or pharmacological treatments. Additionally, given the naturalistic design, we decided not to conduct interim analyses as they might compromise the integrity of the final data analysis. However, we remain attentive to participant safety and feedback. If any adverse events are reported or significant concerns are raised during participant evaluations (as part of Patient and Public Involvement), appropriate steps, including potential study termination, will be considered and documented accordingly.

### Methods for additional analyses (e.g., subgroup analyses) {20b}

Exploratory and demographic variables will be explored for potential associations with the outcome variables. If deemed appropriate, analyses regarding treatment effects on primary and secondary outcomes will be re-evaluated to control for potential confounding effects.

### Methods in analysis to handle protocol non-adherence and any statistical methods to handle missing data {20c}

Multiple imputations will be utilized to address missing data for intention-to-treat (ITT) analyses.

### Plans to give access to the full protocol, participant-level data, and statistical code {31c}

The full study protocol, statistical code, and dataset will be available from the corresponding author upon request.

## Oversight and monitoring

### Composition of the coordinating center and trial steering committee {5d}

The trial is coordinated by the core author group, consisting of the corresponding author and co-authors. Three student research assistants provide support for day-to-day tasks, including participant communication, administrative duties, and data maintenance. The core author group will meet weekly to oversee the trial process. During these meetings, the core author group will discuss the study progress, address challenges, and ensure smooth trial conduct. On a quarterly basis, a current status report is submitted to the relevant stakeholder group, detailing the status of the project, its progress, and any problems that might have arisen. The stakeholder group comprises ten licensed psychotherapists who do not conduct the smoking cessation. As confirmed through the University of Siegen’s ethics committee, the trial can be defined as moderate scale and of low-risk nature. This is why it was decided not to form a formal trial steering committee. The trial is funded by the University of Siegen’s research unit; there is no other funding party involved.

### Composition of the data monitoring committee, its role and reporting structure {21a}

No data monitoring committee has been deemed necessary, as this trial is classified as a low-risk intervention.

### Adverse event reporting and harms {22}

Nicotine withdrawal can, in some cases, lead to increased irritability, sleep disturbances, concentration problems, or heightened feelings of hunger. These withdrawal symptoms typically occur in the first few days after smoking cessation and usually subside within 2 to 3 weeks [15]. These topics will be discussed during the smoking cessation program (sessions 1 and 2), and measures will be collaboratively developed for them. For example, relaxation exercises to cope with irritability, adopting sleep hygiene practices or providing alternatives to manage increased hunger, such as drinking water, consuming sugar-free gum, or engaging in physical activity, will be addressed. No additional mental strains beyond those typical of quitting smoking are expected as a result of the study. Patients who report adverse events will have the option to undergo evaluation by the research staff and a psychotherapist. Oversight of safety, protocol adherence, study quality, and ethical conduct will be the responsibility of the study coordination unit.

### Frequency and plans for auditing trial conduct {23}

ES and MN are responsible for data monitoring. Additionally, trial conduct is regularly monitored through weekly meetings involving the authors, trained psychologists, and research assistants to review study progress, address challenges, and ensure adherence to the trial protocol. Any deviations or issues are promptly documented and discussed. Additionally, internal audits within the core author group of the trial are conducted every 6 months to further ensure compliance and data.

### Plans for communicating important protocol amendments to relevant parties (e.g., trial participants, ethical committees) {25}

Any significant alterations to the protocol that could affect the study’s implementation will be communicated to the stakeholder group and to the University of Siegen’s ethics committee for re-evaluation and approval. Once approval is obtained, the corresponding author will notify the stakeholder group, the primary working group, and the research assistants of the revisions. Participants will also be informed of these changes. Any deviations from the protocol will be fully documented to ensure transparency and proper tracking. Additionally, the updated protocol will be reflected in the clinical trial registry, and the published protocol will be revised accordingly.

### Dissemination plans {31a}

It is planned to publish the results of the trial in a peer-reviewed journal. Additionally, abstracts will be submitted to suitable congresses. Participants will be provided a summary of the trial’s results upon request.

## Discussion

The current study aims to examine the potential benefits of incorporating smoking cessation interventions into standard outpatient psychotherapeutic consultations, considering nicotine addiction a significant health concern. While patients with other substance use disorders in Germany are required to achieve abstinence within the initial ten sessions of psychotherapy for the psychotherapy to be covered by health insurance, tobacco smoking remains unaddressed in psychotherapy sessions, despite existing evidence of nicotine’s negative effect on mental health [13, 52] and its potential to inhibit responsiveness to psychotherapy. Although existing research emphasizes the favorable impact of smoking cessation on mental health, its practical integration into psychotherapeutic settings remains ambiguous.

Although the current state of research suggests that promising outcomes regarding the effectiveness of integrating smoking cessation into psychotherapy are likely, it is essential to investigate potential working mechanisms and necessary conditions for positive outcomes. One notable issue is the possibility of motivational factors influencing participant engagement and outcomes. In the context of psychotherapeutic care research, we decided not to limit participation based on the willingness to quit smoking. This enabled us to examine the effects of actively addressing smoking behavior through interventions on smoking behavior, dependence severity, and mental health markers. Since existing research underscores the mental health benefits of successful smoking cessation, our findings regarding mental health outcomes may be less pronounced, as not all participants may successfully achieve abstinence during the intervention period. Secondary analyses of motivation and self-efficacy will be performed to detect confounding effects on the primary outcome.

The generalizability of our findings may be constrained by the specific characteristics of our sample population and the setting in which the study will be conducted. The study will be carried out in a single outpatient psychotherapeutic clinic, limiting the diversity of participants and potentially affecting the applicability of our findings to other settings or populations.

Nonetheless, we expect to provide novel information on an integrative approach to smoking cessation in the field of psychotherapy, highlighting the potential importance of addressing smoking cessation as part of comprehensive mental health treatment.

If a relationship is found between the intervention and improved symptom outcomes could have significant implications for future research. First, the findings may shed light on the previously overlooked role of smoking within psychotherapeutic practice, potentially explaining instances of nonresponse or slow therapeutic progress. Understanding the impact of smoking on treatment outcomes could inform more comprehensive therapeutic approaches that address smoking cessation alongside mental health management. Second, emphasizing the importance of taking smoking more seriously in the context of mental health treatment could lead to a reevaluation of current practices. This underscores the need for integrated interventions that prioritize smoking cessation alongside traditional psychotherapeutic modalities. Thus, future research should consider the integration of smoking cessation initiatives into or next to psychotherapeutic interventions to optimize treatment effectiveness and enhance overall patient outcomes.

## Trial status

The current trial was prospectively registered in the ISRCTN registry for current controlled trials on 01.05.2024 (ISRCTN12859609, protocol version number 1.0). The recruitment started in May 2024 and the recruitment process is planned to be completed by May 2025. Data collection started in May 2024 and will end by April 2027. This is study protocol version 1, 04.06.2024.

## Data Availability

It is planned to publish the results of the trial in a peer-reviewed journal. Additionally, the final trial dataset will be provided upon request.

## Abbreviations

ANOVA: Analysis of variance
APOI: Attitudes toward Psychological Online Interventions
ASSIST: Alcohol, Smoking, and Substance Involvement Screening Test
BDI-II: Beck Depression Inventory-Revised
BfArM: German Federal Institute for Drugs and Medical Devices
BSCL: Brief Symptom Checklist
CBT: Cognitive behavioral therapy
CO: Carbon monoxide
FEVER: University of Rhode Island Change Assessment Scale
FTND: Fagerström Test for Nicotine Dependence
GSE: Generalized Self-Efficacy Scale
ICD-10: International Statistical Classification of Diseases and Related Health Problems
ITT: Intention-to-treat
MTSS: Motivation to Stop Scale
ROC_AUC_: Area under the receiver operating characteristics
SESS: Self-Efficacy Scale for Smoking
SOC: Scale for Stages of Change
TAU: Treatment a usual
WHO: World Health Organization
WHO-5: WHO-5 Well-being Index
WHOQOL-BREF: WHO Quality of Life Brief Questionnaire
